# Exposure to oxLDL impairs TGF-β activity in human tendon cells

**DOI:** 10.1186/s12891-023-06308-x

**Published:** 2023-03-16

**Authors:** Rouhollah Mousavizadeh, Charlie M. Waugh, Erin DeBruin, Robert G. McCormack, Vincent Duronio, Alex Scott

**Affiliations:** 1 Department of Physical Therapy, Faculty of Medicine, The University of British Columbia, Vancouver, Canada; 2 Department of Orthopaedics, Faculty of Medicine, The University of British Columbia, Vancouver, BC Canada; 3 Department of Medicine, Faculty of Medicine, The University of British Columbia, Vancouver, Canada

**Keywords:** Tendon, oxLDL, TGF-β, Collagen matrix, Hyperlipidemia

## Abstract

**Background:**

Previous studies have shown that patients with hypercholesterolemia experience elevated levels of oxidized LDL (oxLDL), a molecule which triggers inflammation and collagenase activity. In this study we discovered novel mechanistic effects of oxLDL on tendon cells and the mediators regulating matrix remodeling by analyzing the expression and activity of related proteins and enzymes. These effects may contribute to tendon damage in patients with high cholesterol.

**Methods:**

Isolated human tendon cells (male and female donors age 28 ± 1.4 age 37 ± 5.7, respectively) were incubated in the presence or absence of oxLDL. The influence of oxLDL on the expression level of key mRNA and proteins was examined using real time quantitative PCR, ELISA and Western blots. The activities of enzymes relevant to collagen synthesis and breakdown (lysyl oxidase and matrix metalloproteinases) were quantified using fluorometry. Finally, the isolated human tendon cells in a 3D construct were exposed to combinations of oxLDL and TGF-β to examine their interacting effects on collagen matrix remodeling.

**Results:**

The one-way ANOVA of gene expression indicates that key mRNAs including *TGFB, COL1A1, DCN, and LOX* were significantly reduced in human tendon cells by oxLDL while *MMP*s were increased. The oxLDL reduced the activity of LOX at 50 µg/ml, whereas conversely MMP activities were induced at 25 µg/ml (P ≤ 0.01). COL1A1 synthesis and TGF-β secretion were also inhibited (P ≤ 0.05). Adding recombinant TGF-β reversed the effects of oxLDL on the expression of collagens and *LOX*. OxLDL also impaired collagen matrix remodeling (P ≤ 0.01), and adding TGF-β restored the native phenotype.

**Conclusion:**

Exposure to oxLDL in patients with hypercholesterolemia may adversely affect the mechanical and structural properties of tendon tissue through a direct action of oxLDL on tendon cells, including impairment of TGF-β expression. This impairment leads to disturbed matrix remodeling and synthesis, thereby potentially leading to increased risk of acute or chronic tendon injury. Our discovery may provide an opportunity for developing effective treatments for tendon injury in hypercholesterolemia patients by targeting the TGF-β pathway.

**Supplementary Information:**

The online version contains supplementary material available at 10.1186/s12891-023-06308-x.

## Background

Hypercholesterolemia is associated with tendon pathologies [1–3], tendon injury prevalence [4–6] and impaired tendon healing [7]. However, there has been little research into the mechanisms by which cholesterol causes tendons to weaken. The mechanisms of lipid deposition in tendon have been speculated [8]; it is thought that low-density lipoproteins (LDLs) derived from plasma become trapped in the tendon matrix. Subsequent oxidative modification of LDL to oxLDL activates resident tissue macrophages which internalize oxLDL and transform them into foam cells. Accumulation of oxLDL in patients with hypercholesteremia triggers inflammation and expression of metalloproteinases (MMPs) in atherosclerosis [9, 10]. High cholesterol triggers a cascade of inflammatory pathways through inducing reactive oxygen species (ROS) generation and oxidative stress in tendon progenitor cells which diminished expression of tendon markers [11, 12]. Therefore, targeting inflammatory factors and oxidative stress pathways has been suggested as a treatment for preventing tendon injuries in hypercholesterolemia patients [13]. Administration of antioxidant agents in animal models suppress tendon degeneration and inflammation [14]. A few preclinical studies also show the effect of ascorbic acid on improving tendon healing via reducing oxidative stress. However the clinical studies did not support similar results [15]. Also, our unpublished data did not show the efficacy of ascorbic acid in preventing the effects of oxLDL on expression of ECM proteins and enzymes in human tendon cells.

Using cholesterol lowering medication, such as statins, is very effective for treating hypercholesterolemia and improving life expectancy by reducing the risk of related diseases. However, the effects of statins on tendons are controversial [16, 17] and may not promote healing of injured tendons. Therefore, understanding the mechanistic effects of hypercholesterolemia on tendons and the nature of damages is important for providing proper treatments.

We have previously found that mice fed a high-fat diet for 30 weeks demonstrated oxLDL deposition in tendon ECM, and that these tendons had reduced biomechanical strength compared to those on a normal diet [6]. These findings suggest a direct link between hypercholesterolemia, intratendinous oxLDL accumulation and tendon health. At the cellular level, human tenocytes cultured with oxLDL-supplemented media demonstrated increased MMP2 gene expression and decreased collagen (COL1A1, COL3A1) gene expression compared to controls [18]. Inflammation, changes in MMP activity and collagen turnover are therefore proposed as relevant pathogenic mechanisms of hypercholesterolemia on tendon pathology [19].

**Fig. 1:**
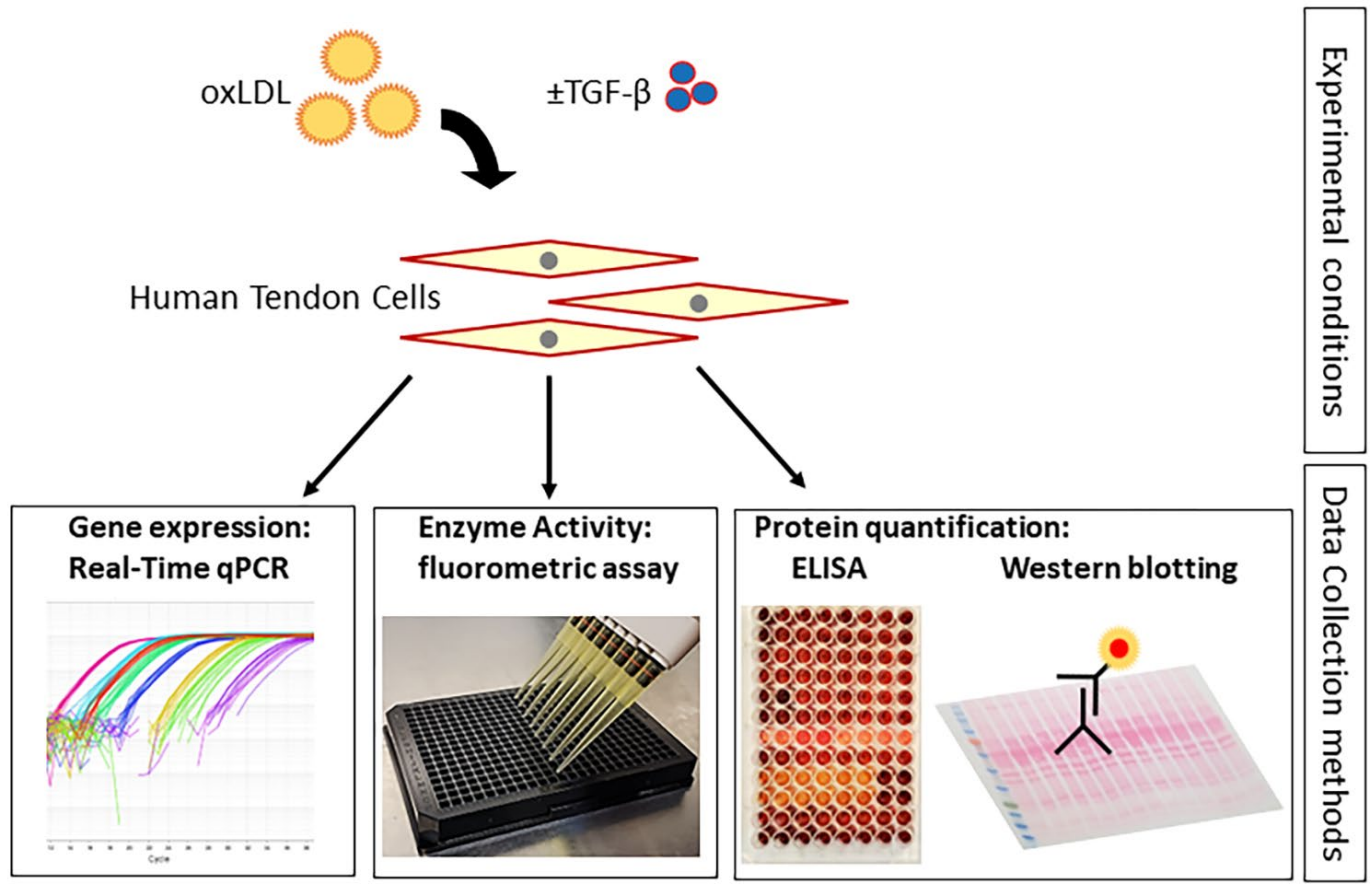
Graphic illustration of the experiment design and data collection methods

Overall, previous studies have indicated the changes in expression of inflammatory factors, matrix proteins and degrading enzymes in association with elevated cholesterol conditions [19]. Yet they did not uncover a mechanism that links high cholesterol to these changes. In this study we sought to investigate the role of oxLDL in tendon pathophysiology more fully by further investigating regulatory pathways that influence matrix remodeling and synthesis in human tendon cells. Specifically, we examined the expression, activity and synthesis of genes, enzymes and proteins relating to matrix synthesis and remodeling after exposure to oxLDL (Fig. 1). Surprisingly, this appears to be the first study that has examined the effect of oxLDL on LOX in tendon cells. We further investigated the impact of oxLDL on a TGF-β and its regulatory effect on LOX and matrix proteins.

## Methods

### Cell culture

Human tendon cells were isolated from healthy human hamstring (semitendinosus) tendons (excess anterior cruciate ligament autograft material) as previously described [20]. Male (N = 2; age 28 ± 1.4) and female (N = 2; age 37 ± 5.7) donors provided written informed consent and the protocol was approved by University of British Columbia Clinical Research Ethics Board (certificate number H10-00220). Equal number of females and males were used in each experiment to exclude the effect of sex as a factor. The tendon cells were dissociated from the tissue using mechanical (chopping with surgical scissors) enzymatic (collagenase) methods. To expand the tendon cell culture, the isolated tendon cells were grown in high glucose Dulbecco’s modified Eagle’s medium (DMEM) supplemented with 10% calf serum, 2 mm L-glutamine, and 1x Antibiotic/Antimycotic solution in a humidified incubator containing 5% CO2 at 37 °C. Then, 10% calf serum was replaced with 5% Lipoprotein Deficient Serum from fetal calf (Sigma, USA, #S5394) before and during incubation with oxLDL. Recombinant TGF-β protein (PeproTech, USA, #100 − 21) at concentration of 5ng/ml was added during the incubation with oxLDL.

### Bioartificial tendon (BAT)

BATs were prepared as previously described [21] with some modifications. Human tendon cells were seeded in the mixture of Purecol EZ Gel (Sigma-Aldrich, USA, # 5074), 5× DMEM, and 5% Lipoprotein Deficient Serum from fetal calf (Sigma, USA, #S5394) with or without 50 µg/ml oxLDL and 5 ng/ml TGF-β. The cells were suspended in the media and other components. Then they were mixed with the neutralized Purecol EZ Gel. The mixture was pipetted between the anchor stems of untreated Tissue Train plate (Flexcell International Corp., USA, #TT-5001U) in each well. After setting the BATs in the incubator, 3ml 1x DMEM media with or without oxLDL and TGF-β was added to each well and the plate was incubated for 72 h (3 days) as the changes in collagen gel by the seeded cells are normally evident within 3 days [22, 23]. The BATs were then scanned using an image scanner and the images were analyzed in ImageJ to measure the diameter of the BATs.

### oxLDL preparation

Isolated Human Low Density Lipoprotein (LDL) (Lee Biosolutions, USA, #360 − 10) was oxidized as previously described [18]. LDL was diluted (200 µg/ml) in 20 ml DPBS containing 5 µM copper sulfate and incubated at 37 °C for 24 h. The oxidation was stopped by adding 1 mM EDTA. The oxLDL was washed and concentrated using Amicon Ultra centrifugal filter unit with the pore size of 10 kDa MWCO (Millipore, USA, #UFC901024). The concentration of oxLDL was measured by a bicinchoninic acid protein assay kit. The concentrated oxLDL was aliquoted and stored in -80 °C.

**Gene expression analysis**: The changes in mRNA expression was analyzed using Real-Time Quantitative Reverse Transcription PCR (RT-qPCR) as previously described [24]. Total RNA was isolated and transcribed to cDNA using Monarch® Total RNA Miniprep Kit (NEB, USA, # T2010S), and High Capacity cDNA Reverse Transcription Kit (Applied Biosystems, USA, #4,368,814), respectively. The oligo primers (Table 1) were custom designed with the PrimerQuest Tool and synthesized by Integrated DNA Technologies (USA). The real-time qPCR reaction was performed using 7500 Fast Real-Time PCR System (Applied Biosystems, USA) and Luna Universal qPCR Master Mix (New England Biolabs, USA, #M3003). The PCR steps were programmed as followed: 95 °C for 1 min, followed by 45 cycles at 95 °C for 15 s and 60 °C for 30 s. ΔCt values were calculated as Ct _Target_ – Ct _GAPDH_) and used for graphs and statistical analysis.

**Table 1:** The sequences of oligo DNA used as primer for qRT-PCR

Gene	Forward primer	Reverse primer
COL1A1	CACCAATCACCTGCGGTACAGAA	CAGATCACGTCATCGCACAAC
COL1A2	AGAGTGGAGCAGTGGTTACTA	GATACAGGTTTCGCCAGTAGAG
COL3A1	AATCAGGTAGACCCGGACGA	TTCGTCCATCGAAGCCTCTG
DCN	GTCACAGAGCAGCACCTACC	TTGTCCAGACCCAAATCAGAACA
ILK	CCCACGACATGCACTCAATA	GACCAGGACATTGGAAAGAGAA
ITGB1	TGATCCTGTGTCCCATTGTAAG	TGACCTCGTTGTTCCCATTC
LOX	TACCCAGCCGACCAAGATA	TGGCATCAAGCAGGTCATAG
MMP1	CAGAAAGAGACAGGAGACATGAG	GAAGAGTTATCCCTTGCCTATCC
MMP2	AGAGAACCTCAGGGAGAGTAAG	CCTCGAACAGATGCCACAATA
MMP3	GTGAGGACACCAGCATGAA	GACCACTGTCCTTTCTCCTAAC
MMP8	GGGTGGGCTCTAAATCCATTAT	CCAATCTCTGCCTCTGTCTTC
MMP9	GAACTTTGACAGCGACAAGAAG	CGGCACTGAGGAATGATCTAA
MMP10	GGCCCTCTCTTCCATCATATTT	CCTGCTTGTACCTCATTTCCT
MMP13	AGCATCTGGAGTAACCGTATTG	CCCGCACTTCTGGAAGTATT
MMP14	GCCGACTAAGCAGAAGAAAGA	TGTCGGCTTGGAGTTAAAGG
TGFB1	CCTGCCTGTCTGCACTATTC	TGCCCAAGGTGCTCAATAAA
GAPDH	TCTTTTGCGTCGCCAGCCGAG	TGACCAGGCGCCCAATACGAC

### Enzymatic assays

To evaluate the activity of LOX and MMPs in protein lysate of human tendon cells, fluorometric assay kits were used. According to the MMP activity assay kit (Abcam, USA, #ab112146), protein lysates were mixed with 4-aminophenylmercuric acetate (APMA) and incubated at 37 °C for 3 h to activate MMPs. Then, MMP green substrate solution was added and incubated for 30 min. The fluorescence intensity was measured at 490/525 nm (Ex/Em) using a microplate reader (Infinite F500, Tecan, Austria). Amplite™ Fluorimetric Lysyl Oxidase Assay kit (AAT Bioquest, USA, #15,255) was used according to the manual to measure LOX activity in protein lysate. The recorded relative fluorescence units from the MMP and LOX assay kits were normalized to the total protein concentrations measured by BCA assay.

**Protein quantification**: The secretion of TGF-β1 was quantified in the conditioned media of human tendon cells using an ELISA kit (R&D System, USA, #DY246) according to the manual. The level of COL1A1 protein in cell lysate was measured using western blot as previously described [24]. Total proteins were resolved and transferred to a 0.45 μm nitrocellulose membrane followed by blocking with 5% BSA. The membrane was probed with COL1A1 (Cell Signaling Technology, USA, #84,336) and Vinculin (Sigma-Aldrich, USA, #v9131). The western blots of COL1A1and Vinculin were detected by IRDye 680 and HRP-conjugated antibodies, then visualized using the G:BOX Chemi XT4 Gel Documentation System (Syngene, UK) and Odyssey CLx Imaging System (LI-COR, USA) with default settings, respectively.

### Statistics

All data was analyzed by either paired t-test or one-way ANOVA followed by Dunnett’s multiple comparison test. GraphPad prism version 7.03 was used to analyze data and generate graphs. The number of biological replicates is reported in the figure captions. *P* values of less than 0.05 were regarded as statistically significant.

## Results

### oxLDL changes the expression of collagen and the factors that mediate matrix remodeling

**Fig. 2:**
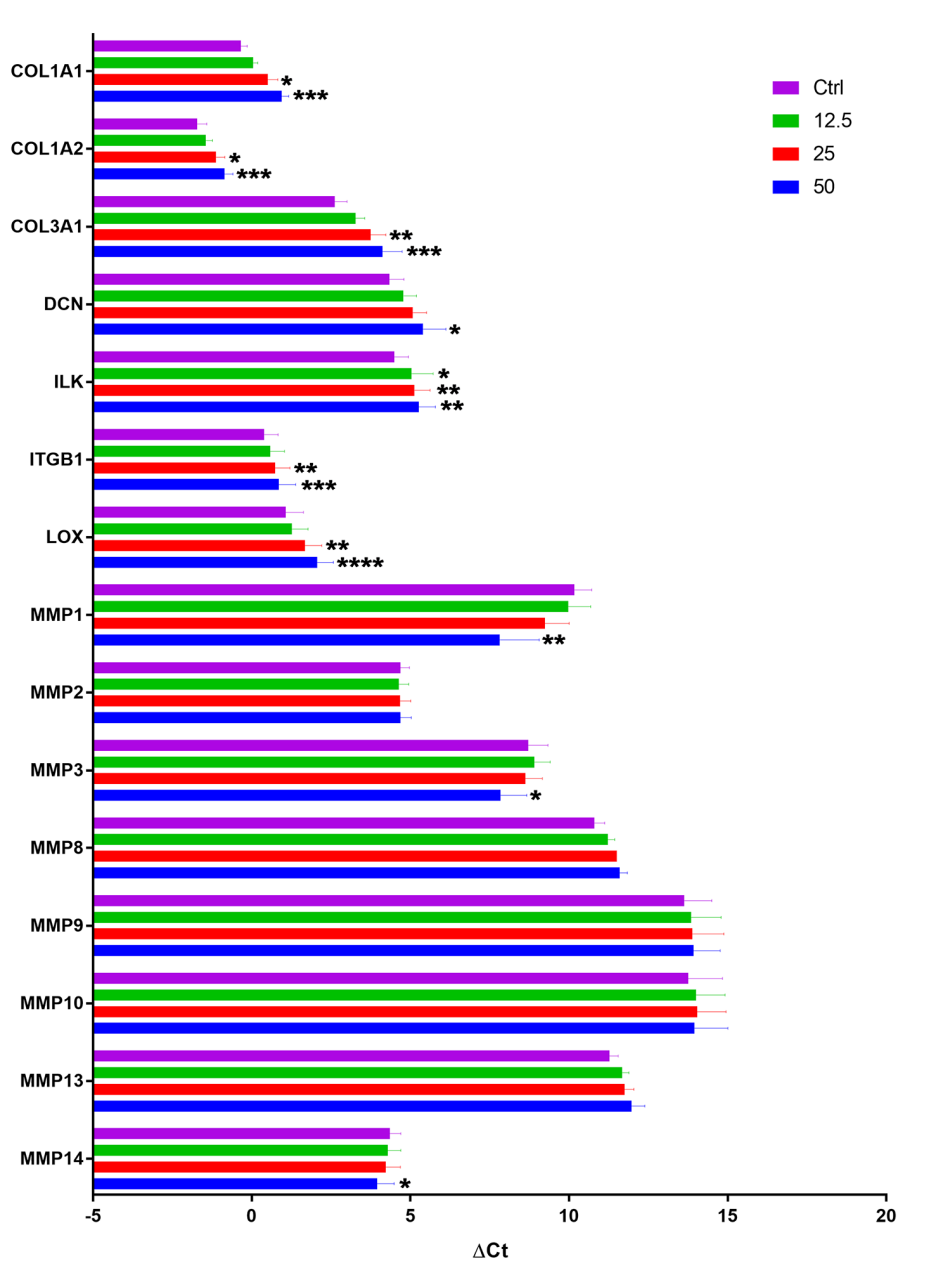
oxLDL changes the expression of collagen and the factors that mediate matrix remodeling. Exposure of human tendon cells to progressively increasing doses of oxLDL resulted in reduction of key mRNAs involved in anabolic processes such as *COL1A1*, *COL1A2, COL3A1, DCN, ILK, ITGB1*, and *LOX*. Conversely, the expression of specific MMPs (*MMP1, MMP3*, and *MMP14*) were increased. Mean ± SE; * P ≤ 0.05; **P ≤ 0.01; ***P ≤ 0.001; ****P ≤ 0.0001; n = 4 biological replicates

In order to determine the effect of oxLDL on mRNA expression of other matrix proteins and factors that regulate tendon matrix synthesis and remodeling, we used qRT-PCR. Our data indicate a significant reduction of extracellular matrix mRNA (*COL1A1, COL1A2, COL3A1* and *DCN*) while the expression of *MMPs* (*MMP1, MMP3* and *MMP14*) was increased. *LOX* mRNA also was reduced by oxLDL. The qRT-PCR results showed that oxLDL reduced the expression of *ILK, ITGB1* and *TGF-β*, which mediate collagen synthesis and mechanotransduction [24, 25] (Fig. 2).

### oxLDL changes the activity of MMPs and LOX

**Fig. 3:**
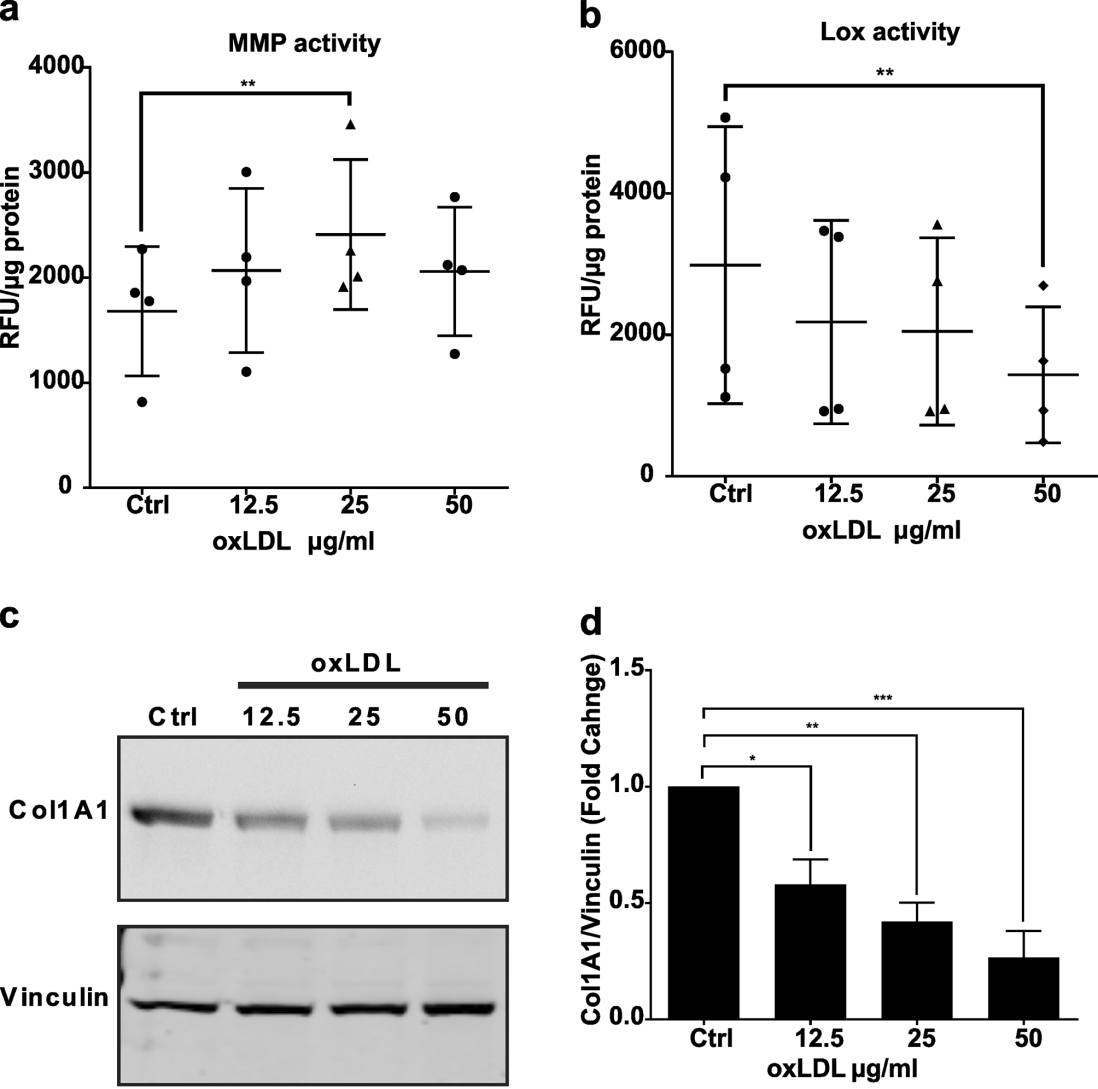
oxLDL changes the activity of enzymes regulating matrix remodeling, and protein expression of collagen. OxLDL increases MMPs activity (**a**) and reduces LOX activity (**b**) in human tendon cells. Western blot shows reductions of COL1A1 protein in human tendon cells after incubation with oxLDL (**c**). The densitometry analysis of Col1A1/Vinculin ratios shows the effect is statically significant and responsive to the dose of oxLDL (**d**). The uncropped images of blots are included in Supplementary information. Mean ± SE; ns P > 0.05; * P ≤ 0.05; **P ≤ 0.01; ***P ≤ 0.001; n = 4 biological replicates

MMPs are degradative enzymes that regulate extracellular matrix remodeling in tendon tissue. Increased activity of MMPs is associated with tendon injuries [26]. The family of LOX enzymes regulate collagen fibrillogenesis by enzymatic cross-linking of collagen matrix, a process which is critical for the proper development of tendon tissue [27, 28]. To measure the activity of LOX and MMPs, we used fluorometric assays that utilize specific synthetic substrates as probes for detection of oxidization and peptide degradation by LOX and MMPs, respectively. Our data indicate oxLDL decreased the activity of LOX in protein lysates of human tendon cells while the MMP activity was increased (Fig. 3a, b).

### oxLDL reduces collagen synthesis by human tendon cells

Collagen type I, as a major component of the tendon matrix, has a pivotal role in the mechanical strength of tendon tissue [29]. We used Western immunoblot to measure the rate of collagen synthesis in incubated human tendon cells with different concentrations of oxLDL. The scans of immunoblots indicate diminished synthesis of COL1A1 protein, the major subunit of collagen type I, in protein lysates of treated human tendon cells with oxLDL (Fig. 3c). The densitometry data show a significant, dose-responsive reduction of COL1A1 protein in the presence of oxLDL (Fig. 3d).

### Reduced expression of TGF-β by oxLDL is attributed to changes in collagen and LOX expression but not MMPs

**Fig. 4:**
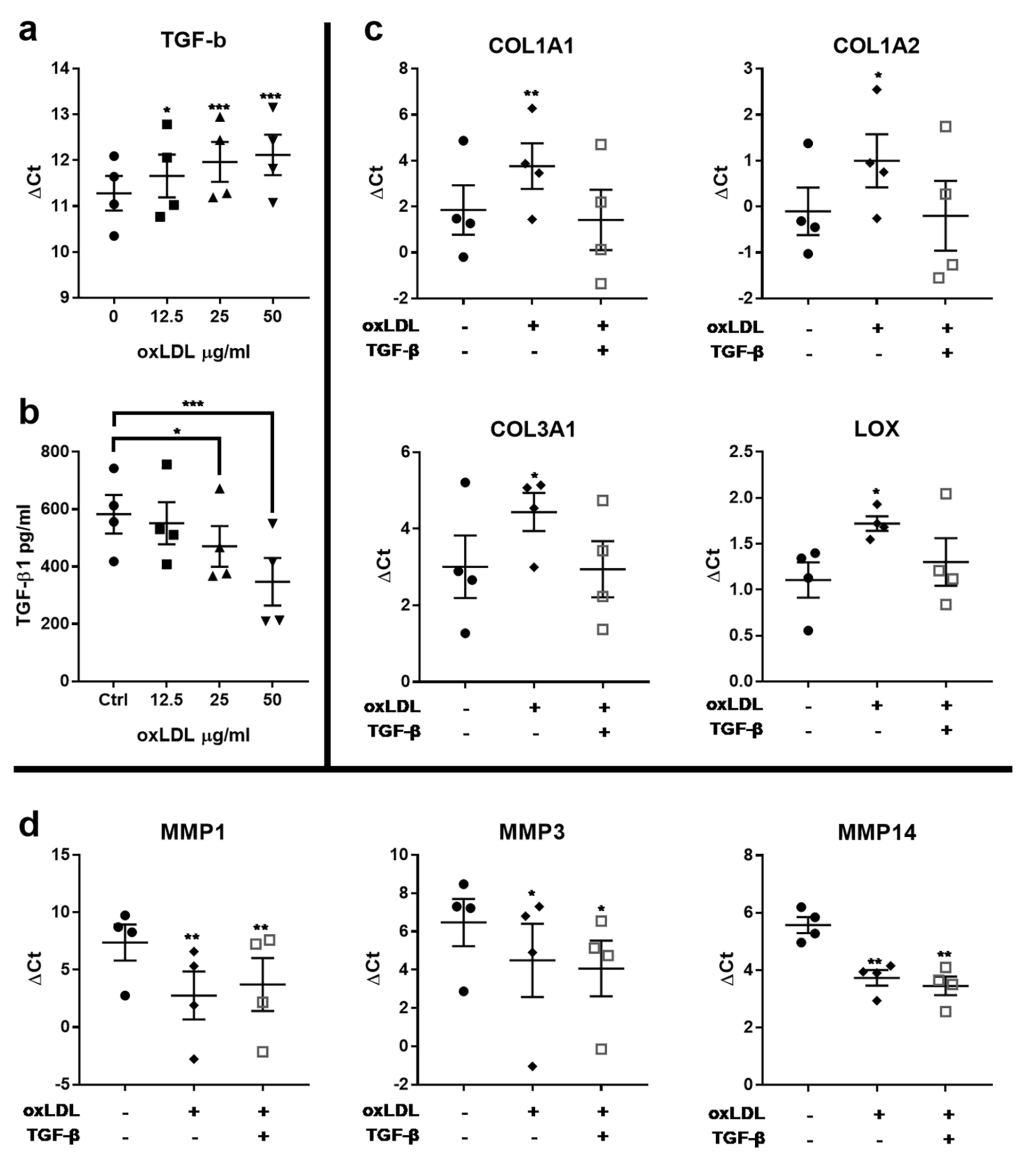
oxLDL changes the expression of collagen and lox via TGF-β. oxLDL decreases TGF-β expression at the level of mRNA (**a**) and protein (**b**). Adding recombinant TGF-β to human tendon cells treated with oxLDL rescued the expression of collagen and *LOX* mRNA (**c**) but not *MMP*s (**d**). Mean ± SE; * P ≤ 0.05; **P ≤ 0.01; ***P ≤ 0.001; n = 4 biological replicates

We examined the possible upstream role of TGF-β in regulating the response of tenocytes to oxLDL, hypothesizing that TGF-β may be responsible for some of the observed changes. The ELISA and qRT-PCR results showed that oxLDL decreases TGF-β expression at the level of mRNA (Fig. 4a) and protein (Fig. 4b). Adding recombinant TGF-β to human tendon cells treated with oxLDL rescued the expression of collagen and *LOX* genes but not *MMP*s (Fig. 4c, d).

### oxLDL disturbs matrix remodeling

**Fig. 5:**
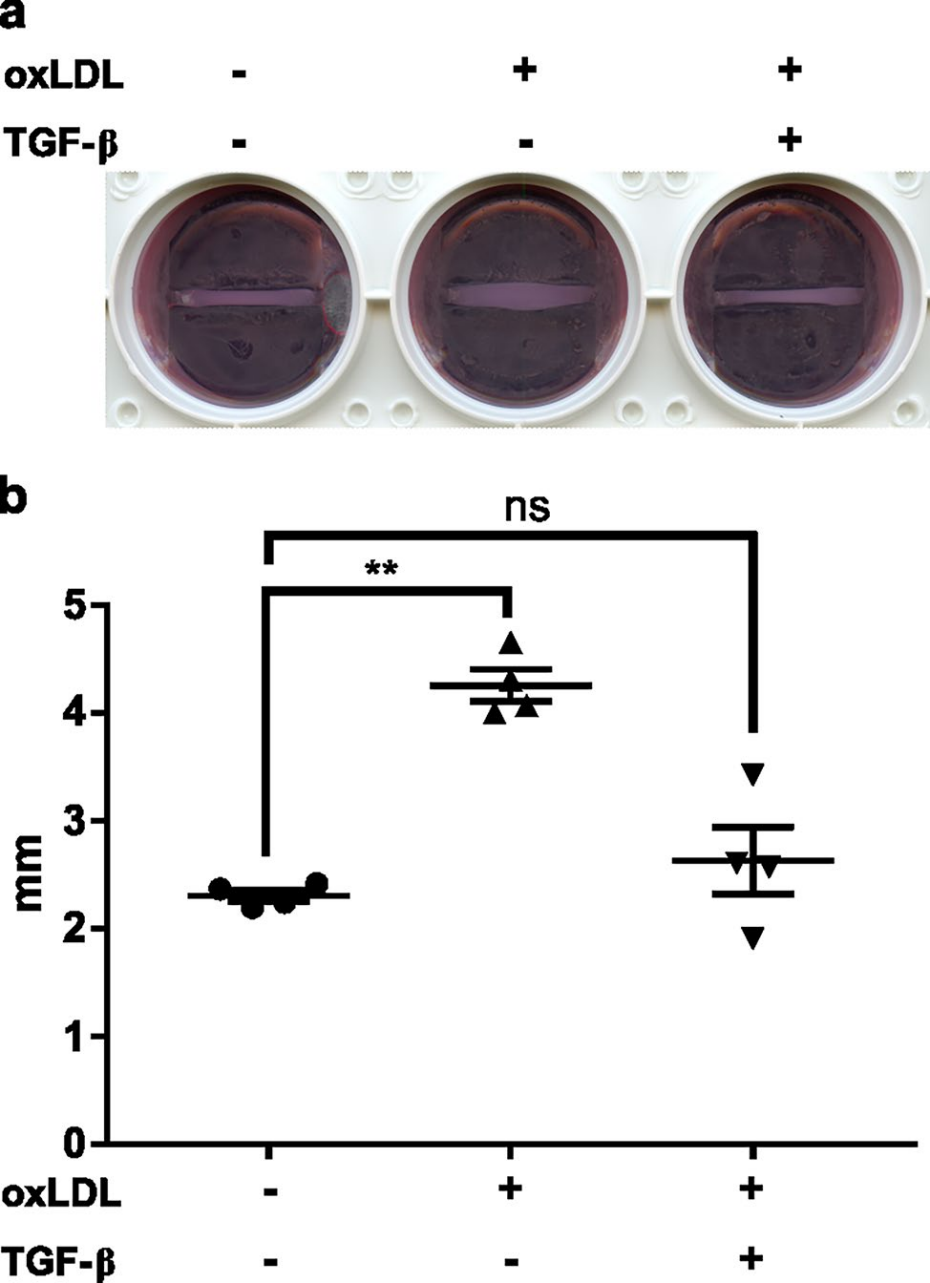
oxLDL disturbed matrix remodeling of BAT via TGF-β. The scanned micrograph of BATs shows impaired BATs remodeling in presence of oxLDL which was reversed by adding recombinant TGF-β (**a**). The impact of oxLDL on thickness of BAT was significant while adding TGF-β abrogated this effect (**b**). Mean ± SE; ns P > 0.05; **P ≤ 0.01; n = 4 biological replicates

Remodeling of the collagen matrix is prominent during tissue development and wound healing [30, 31]. We used a modified BAT composed of human tendon cells in 3D collagen matrix to examine the impact of oxLDL on tendon remodeling. oxLDL disrupted the remodeling of BAT compared to the control while adding recombinant TGF-β rescued the ability of tendon cells for matrix remodeling (Fig. 5a). Measuring the thickness of BATs showed that the effect of oxLDL was significant (Fig. 5b).

## Discussion

This study showed that oxLDL disturbed a number of important but divergent processes in human tendon cells, including the remodeling, synthesis and cross-linking of key tendon extracellular matrix components. The expression and activity of degrading enzymes such as MMP1, MMP3 and MMP14 were increased while LOX expression and activity was reduced. Also, the expression of genes with key functions in the adaptive response to mechanical loading - *ILK, ITGB1* and *TGF-β* - were diminished in human tendon cells by oxLDL. Our results indicate that downregulation of *TGF-β* by oxLDL promotes reduced expression of collagen and *LOX* which may contribute to changes in BAT matrix remodeling, but further unidentified mechanisms must be responsible for the increased MMP activity.

LOX activity is critical for crosslinking of collagen fibrils and proper assembly of the tendon matrix to confer tensile strength to the tendon tissue [32]. Dysregulation of LOX has been reported as the etiology of various disorders, which introduces LOX as a potential therapeutic target [33]. The inhibition of LOX prevents collagen fibril formation by human tendon cells [27], and the treatment of tendon with recombinant LOX improves the mechanical properties of tendon without any changes in collagen synthesis [34]. LOX also promotes cell-mediated collagen contraction due to collagen crosslinking and subsequent increased ECM stiffness [35, 36]. The reduced activity and expression of LOX in human tendon cells by oxLDL may impair collagen fiber formation and remodeling in tendon.

Our study demonstrated a significant effect of oxLDL on the regulation of MMP activity in human tendon cells. This effect, in conjunction with the diminished LOX activity discussed above, may explain the inferior mechanical properties of tendon tissues in animals with high cholesterol [7, 18, 37]. A balance of LOX and MMP is essential for tendon homeostasis, which involves both crosslinking and degradation of the ECM, respectively [38, 39]. The impaired regulation of MMP activity has been suggested to have a major role in the development of tendon injuries [32, 40]. Our gene expression analysis showed increased expression of *MMP-1, MMP-3* and *MMP-14* in human tendon cells by oxLDL but no significant changes in other MMPs (*MMP-2, 8, 9, 13*). MMP1, MMP13 and MMP14 catalyze the degradation of collagen type 1 and 3 [41]. MMP14, known as membrane type 1 MMP (MT1-MMP), also activates pro-MMP2 by its proteolytic cleavage to the mature MMP2 which has collagenolytic activity [42, 43]. The imbalanced activity of MMP can lead to tendon pathology or injuries [44]. The overall pattern of oxLDL increasing specific MMPs has been observed in other cell types as well: oxLDL induces the expression of *MMP9* in human monocyte-derived macrophages while its inhibitor *TIMP1* was reduced [45] whereas in endothelial cells, oxLDL increases *MMP1* expression. Increased activity and expression of MMPs by oxLDL in tendon cells potentially contribute to destruction of tendon matrix by degradation of collagen fibers.

The uptake of LDL participles into cells is regulated by the LDL receptor [46]. However, oxidization of LDL prevents its binding to the receptors [47]. The scavenger receptors, including Lectin-like oxidized LDL receptor-1 (LOX-1); CD36; and scavenger receptor A, can bind to oxLDL to promote its internalization in certain cell types [47]. However, the process of binding or uptake of oxLDL by tendon cells which exerts its effect on tendon tissue has not yet been determined. Our results indicate that oxLDL reduced collagen and *LOX* expression via downregulation of *TGF-β*, which was reversed by adding recombinant TGF-β to the human tendon cell culture.

The remodeling of the collagen matrix, which was disturbed by oxLDL in BATs, is mainly controlled by TGF-β [48, 49]. TGF-β is a key factor in various biological processes that regulate healing, development and mechanical properties of tendon [50–52] including tendon cell migration and collagen synthesis [53]. TGF-β regulates cell-matrix interaction and contractility of the collagen matrix [48]. Changes in collagen organization and contraction require the presence of certain integrins including β1 integrin [49, 54, 55]. Downregulation of TGF-β and β1 integrin by oxLDL can abrogate cell-induced collagen contraction and matrix reorganization in BATs. These events prevent the changes in thickness of BATS which can be rescued by adding TGF-β.

In tendons, tissue adaptation is heavily influenced by physical activity, which is experienced by cells as mechanical forces transmitted from the extracellular matrix to the cells in a continuous feedback process [56]. Integrins and TGF-β are thought to play an important role in this ongoing physiological process [24, 51]. The observed downregulation of integrins, integrin-related proteins (ITGB1 and ILK) and TGF-β in the presence of oxLDL may impair the normal adaptive response to exercise in individuals with hypercholesterolemia.

Our study proposes a mechanistic basis for the etiology of tendon injury in people with hypercholesterolemia. Our study supports previously established detrimental effects of hypercholesterolemia on tendon tissue by revealing a potential mechanism by which oxLDL impairs tendon cells and matrix remodeling. However, this study is limited to the in vitro conditions and may not include other causative factors such as changes in mechanical load of tendons due to different lifestyle and body mass index in patients with hypercholesterolemia. Further studies using animal models and clinical subjects could demonstrate the longer-term effect effects of hypercholesterolemia on tendon matrix and potential role of targeting TGF-beta to minimize or reverse cholesterol-induced tendon changes.

## Conclusion

Overall, this study describes potentially detrimental effects of oxLDL on the homeostasis of tendons, which occurs through direct action of oxLDL on tendon fibroblasts. These effects may be driven by changes in ECM degradation, crosslinking, and synthesis. TGF-β appears to restore collagen crosslinking and synthesis. However, the effect of oxLDL on ECM degrading enzymes is affected by different mechanisms. Further mechanistic studies may unravel novel therapeutic targets for managing tendon injuries in patients with high lipid conditions.

## Electronic supplementary material

Below is the link to the electronic supplementary material.

Supplementary Material 1

Supplementary Material 2

## Data Availability

The RT-qPCR dataset is available in the Supplementary Dataset File. The uncropped images of blots used in Fig. 3c are included as Supplementary Info File. We included individual data points in the graphs wherever was possible.

## List of abbreviations

BAT: Bio-artificial tendon
ECM: extracellular matrix
ILK: Integrin-linked kinase
ITGB1: Integrin Subunit Beta 1
LOX: Lysyl oxidase
MMP: Matrix metalloproteinase
oxLDL: oxidized Low Density Lipoprotein
TGF: Transforming growth factor beta
TIMP: Tissue inhibitor of metalloproteinases

## References

[1] Abboud JA, Kim JS (2010). The Effect of Hypercholesterolemia on Rotator Cuff Disease. Clin Orthop Relat Res.

[2] Ozgurtas T, Yildiz C, Serdar M, Atesalp S, Kutluay T (2003). Is high concentration of serum lipids a risk factor for Achilles tendon rupture?. Clin Chim Acta.

[3] Klemp P, Halland AM, Majoos FL, Steyn K (1993). Musculoskeletal manifestations in hyperlipidaemia: a controlled study. Ann Rheum Dis.

[4] Tilley BJ, Cook JL, Docking SI, Gaida JE (2015). Is higher serum cholesterol associated with altered tendon structure or tendon pain? A systematic review. Br J Sports Med.

[5] Gaida JE, Ashe MC, Bass SL, Cook JL (2009). Is adiposity an under-recognized risk factor for tendinopathy? A systematic review. Arthritis Rheum.

[6] Scott A, Zwerver J, Grewal N, de Sa A, Alktebi T, Granville DJ (2015). Lipids, adiposity and tendinopathy: is there a mechanistic link? Critical review. Br J Sports Med.

[7] Beason DP, Tucker JJ, Lee CS, Edelstein L, Abboud JA, Soslowsky LJ (2014). Rat rotator cuff tendon-to-bone healing properties are adversely affected by hypercholesterolemia. J Shoulder Elbow Surg.

[8] Oosterveer DM, Versmissen J, Yazdanpanah M, Defesche JC, Kastelein JJP, Sijbrands EJG (2010). The risk of tendon xanthomas in familial hypercholesterolaemia is influenced by variation in genes of the reverse cholesterol transport pathway and the low-density lipoprotein oxidation pathway. Eur Heart J.

[9] Tekin IO, Orem A, Shiri-Sverdlov R (2013). Oxidized LDL in inflammation: from bench to bedside. Mediators Inflamm.

[10] Pirillo A, Norata GD, Catapano AL (2013). LOX-1, OxLDL, and atherosclerosis. Mediat Inflamm.

[11] Li K, Deng G, Deng Y, Chen S, Wu H, Cheng C (2019). High cholesterol inhibits tendon-related gene expressions in tendon-derived stem cells through reactive oxygen species-activated nuclear factor-κB signaling. J Cell Physiol.

[12] Fang WH, Bonavida V, Agrawal DK, Thankam FG (2023). Hyperlipidemia in tendon injury: chronicles of low-density lipoproteins. Cell Tissue Res.

[13] Yazdani AN, Rai V, Agrawal DK (2022). Rotator Cuff Health, Pathology, and repair in the perspective of hyperlipidemia. J Orthop Sports Med.

[14] Lui PPY, Zhang X, Yao S, Sun H, Huang C (2022). Roles of oxidative stress in Acute Tendon Injury and degenerative Tendinopathy-A target for intervention. Int J Mol Sci.

[15] DePhillipo NN, Aman ZS, Kennedy MI, Begley JP, Moatshe G, LaPrade RF (2018). Efficacy of vitamin C supplementation on collagen synthesis and oxidative stress after Musculoskeletal Injuries: a systematic review. Orthop J Sports Med.

[16] Eliasson P, Dietrich-Zagonel F, Lundin A-C, Aspenberg P, Wolk A, Michaëlsson K (2019). Statin treatment increases the clinical risk of tendinopathy through matrix metalloproteinase release – a cohort study design combined with an experimental study. Sci Rep.

[17] de Sá A, Hart DA, Khan K, Scott A (2018). Achilles tendon structure is negatively correlated with body mass index, but not influenced by statin use: a cross-sectional study using ultrasound tissue characterization. PLoS ONE.

[18] Grewal N, Thornton GM, Behzad H, Sharma A, Lu A, Zhang P (2014). Accumulation of oxidized LDL in the Tendon Tissues of C57BL/6 or apolipoprotein E knock-out mice that consume a high Fat Diet: potential impact on Tendon Health. PLoS ONE.

[19] Yang Y, Lu H, Qu J (2018). Tendon pathology in hypercholesterolaemia patients: epidemiology, pathogenesis and management. J Orthop Translat.

[20] Mousavizadeh R, Khosravi S, Behzad H, McCormack RG, Duronio V, Scott A (2014). Cyclic strain alters the expression and release of angiogenic factors by human tendon cells. PLoS ONE.

[21] Scott A, Danielson P, Abraham T, Fong G, Sampaio AV, Underhill TM (2011). Mechanical force modulates scleraxis expression in bioartificial tendons. J Musculoskelet Neuronal Interact.

[22] Schulz Torres D, Freyman M, Yannas T, Spector IV (2000). Tendon cell contraction of collagen–GAG matrices in vitro: effect of cross-linking. Biomaterials.

[23] Qi J, Chi L, Faber J, Koller B, Banes AJ (2007). ATP reduces gel compaction in osteoblast-populated collagen gels. J Appl Physiol.

[24] Mousavizadeh R, Hojabrpour P, Eltit F, McDonald PC, Dedhar S, McCormack RG (2020). β1 integrin, ILK and mTOR regulate collagen synthesis in mechanically loaded tendon cells. Sci Rep.

[25] Maeda T, Sakabe T, Sunaga A, Sakai K, Rivera AL, Keene DR (2011). Conversion of Mechanical Force into TGF-β-Mediated biochemical signals. Curr Biol.

[26] Del Buono A, Oliva F, Osti L, Maffulli N (2013). Metalloproteases and tendinopathy. Muscles Ligaments Tendons J.

[27] Herchenhan A, Uhlenbrock F, Eliasson P, Weis M, Eyre D, Kadler KE (2015). Lysyl Oxidase activity is required for ordered collagen fibrillogenesis by Tendon cells. J Biol Chem.

[28] Marturano JE, Arena JD, Schiller ZA, Georgakoudi I, Kuo CK (2013). Characterization of mechanical and biochemical properties of developing embryonic tendon. Proc Natl Acad Sci U S A.

[29] Liu SH, Yang RS, al-Shaikh R, Lane JM. Collagen in tendon, ligament, and bone healing. A current review. Clin Orthop Relat Res. 1995;:265–78. 7671527

[30] Bell E, Ivarsson B, Merrill C (1979). Production of a tissue-like structure by contraction of collagen lattices by human fibroblasts of different proliferative potential in vitro. PNAS.

[31] Stopak D, Harris AK (1982). Connective tissue morphogenesis by fibroblast traction: I. tissue culture observations. Dev Biol.

[32] Magra M, Maffulli N (2005). Matrix metalloproteases: a role in overuse tendinopathies. Br J Sports Med.

[33] Yang N, Cao D-F, Yin X-X, Zhou H-H, Mao X-Y (2020). Lysyl oxidases: emerging biomarkers and therapeutic targets for various diseases. Biomed Pharmacother.

[34] Nguyen PK, Jana A, Huang C, Grafton A, Holt I, Giacomelli M (2022). Tendon mechanical properties are enhanced via recombinant lysyl oxidase treatment. Front Bioeng Biotechnol.

[35] Barker HE, Bird D, Lang G, Erler JT. Tumor-secreted LOXL2 activates fibroblasts through FAK Signaling. Mol Cancer Res. 2013;11. 10.1158/1541-7786.MCR-13-0033-T. PMC3833835 24008674

[36] de Jong OG, van Balkom BWM, Gremmels H, Verhaar MC (2016). Exosomes from hypoxic endothelial cells have increased collagen crosslinking activity through up-regulation of lysyl oxidase‐like 2. J Cell Mol Med.

[37] Steplewski A, Fertala J, Tomlinson R, Hoxha K, Han L, Thakar O (2019). The impact of cholesterol deposits on the fibrillar architecture of the Achilles tendon in a rabbit model of hypercholesterolemia. J Orthop Surg Res.

[38] Xiao Q, Ge G (2012). Lysyl Oxidase, Extracellular Matrix Remodeling and Cancer Metastasis. Cancer Microenviron.

[39] Page-McCaw A, Ewald AJ, Werb Z (2007). Matrix metalloproteinases and the regulation of tissue remodelling. Nat Rev Mol Cell Biol.

[40] Minkwitz S, Schmock A, Kurtoglu A, Tsitsilonis S, Manegold S, Wildemann B (2017). Time-dependent alterations of MMPs, TIMPs and Tendon structure in Human Achilles Tendons after Acute rupture. Int J Mol Sci.

[41] Fields GB (2013). Interstitial collagen catabolism. J Biol Chem.

[42] Holmbeck K, Bianco P, Caterina J, Yamada S, Kromer M, Kuznetsov SA (1999). MT1-MMP-deficient mice develop dwarfism, osteopenia, arthritis, and connective tissue disease due to inadequate collagen turnover. Cell.

[43] Aimes RT, Quigley JP (1995). Matrix metalloproteinase-2 is an interstitial collagenase. Inhibitor-free enzyme catalyzes the cleavage of collagen fibrils and soluble native type I collagen generating the specific 3/4- and 1/4-length fragments. J Biol Chem.

[44] Davis ME, Gumucio JP, Sugg KB, Bedi A, Mendias CL (2013). MMP inhibition as a potential method to augment the healing of skeletal muscle and tendon extracellular matrix. J Appl Physiol (1985).

[45] Xu XP, Meisel SR, Ong JM, Kaul S, Cercek B, Rajavashisth TB (1999). Oxidized low-density lipoprotein regulates matrix metalloproteinase-9 and its tissue inhibitor in human monocyte-derived macrophages. Circulation.

[46] Goldstein JL, Brown MS (2009). The LDL receptor. Arterioscler Thromb Vasc Biol.

[47] Levitan I, Volkov S, Subbaiah PV, Oxidized LDL (2010). Diversity, patterns of Recognition, and pathophysiology. Antioxid Redox Signal.

[48] Montesano R, Orci L (1988). Transforming growth factor beta stimulates collagen-matrix contraction by fibroblasts: implications for wound healing. PNAS.

[49] Riikonen T, Koivisto L, Vihinen P, Heino J (1995). Transforming growth Factor-β regulates collagen gel contraction by increasing α2β1 integrin expression in osteogenic cells (∗). J Biol Chem.

[50] Tan G-K, Pryce BA, Stabio A, Brigande JV, Wang C, Xia Z (2020). Tgfβ signaling is critical for maintenance of the tendon cell fate. Elife.

[51] Gumucio JP, Sugg KB, Mendias CL (2015). TGF-β superfamily signaling in muscle and tendon adaptation to resistance exercise. Exerc Sport Sci Rev.

[52] Klein MB, Yalamanchi N, Pham H, Longaker MT, Chan J (2002). Flexor tendon healing in vitro: Effects of TGF-β on tendon cell collagen production. J Hand Surg.

[53] Kaji DA, Howell KL, Balic Z, Hubmacher D, Huang AH (2020). Tgfβ signaling is required for tenocyte recruitment and functional neonatal tendon regeneration. Elife.

[54] Burgess ML, Carver WE, Terracio L, Wilson SP, Wilson MA, Borg TK (1994). Integrin-mediated collagen gel contraction by cardiac fibroblasts. Effects of angiotensin II. Circ Res.

[55] Gullberg D, Tingström A, Thuresson AC, Olsson L, Terracio L, Borg TK (1990). Beta 1 integrin-mediated collagen gel contraction is stimulated by PDGF. Exp Cell Res.

[56] Kjaer M (2004). Role of extracellular matrix in adaptation of tendon and skeletal muscle to mechanical loading. Physiol Rev.

