# Supplementary material for: Exposure to oxLDL impairs TGF-β activity in human tendon cells
Source: BMC Musculoskelet Disord. 2023 Mar 16;24:197. doi: 10.1186/s12891-023-06308-x (PMC10018928; doi:10.1186/s12891-023-06308-x)
Supplement: Supplementary file 2 — Supplementary Material 2 [file 12891_2023_6308_MOESM2_ESM.pdf]

Exposure to oxLDL impairs TGF-β activity in human tendon cells

Rouhollah Mousavizadeh[1], Charlie M. Waugh[1], Erin DeBruin[1], Robert G. McCormack[2], Vincent Duronio[3], Alex Scott[1]

1. Department of Physical Therapy, Faculty of Medicine, The University of British Columbia, Vancouver, Canada

2. Department of Orthopaedics, Faculty of Medicine, The University of British Columbia, Vancouver, BC, Canada

3. Department of Medicine, Faculty of Medicine, The University of British Columbia, Vancouver, Canada

Supplementary information

The Uncropped images of blots used in figure 3c:

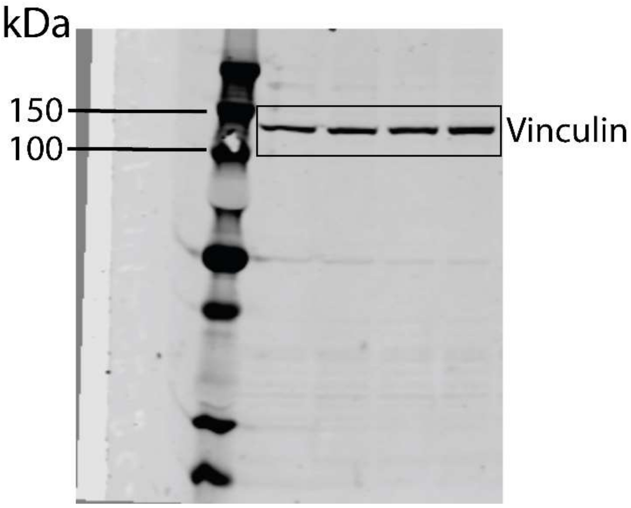

IRDye 680 signal

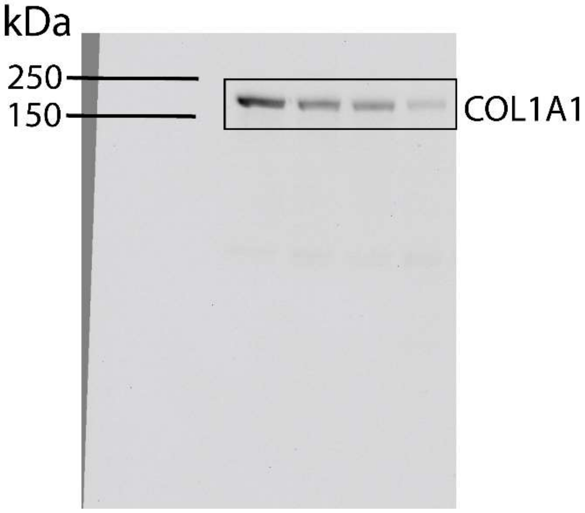

Chemiluminescent signal
